# Next generation of “magic bullets”, solutions from the microbial pangenome

**DOI:** 10.1038/s44321-024-00133-y

**Published:** 2024-09-06

**Authors:** Vega Masignani, Rino Rappuoli, Mariagrazia Pizza

**Affiliations:** 1 GSK, 53100 Siena, Italy; 2 Fondazione Biotecnopolo di Siena, 53100 Siena, Italy; 3 Imperial, Department of Life Science, CBRB, London, SW7 2AZ UK

**Keywords:** Biotechnology & Synthetic Biology, Methods & Resources, Microbiology, Virology & Host Pathogen Interaction

## Abstract

V. Masignani, R. Rappuoli, and M. Pizza discuss the study from Gill et al, published in this issue of *EMBO Mol Med*, that describes a new toxin platform for cancer therapy.

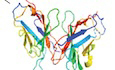

## Teaser

Identification of a toxic agent that—like a sniper—would be capable of targeting pathogenic bacteria and effectively discriminating between “good” and “evil” cells has long been the holy grail of drug discovery. The theory of magic bullet, first pioneered by Paul Ehrlich in the early 1900, has represented since then a “*leit motif”* in immunological research against infectious diseases. Salvarsan, the first arsenic-based drug against syphilis, was the first example showing that this concept was in fact a realistic goal. Later, a paradigm shift took place with pathogenic bacteria becoming the source of the magic bullets (i.e., the toxins), and tumorigenic cells the enemy to combat; thus paving the way to immunotoxins discovery and development. In this issue of *EMBO Molecular Medicine*, Gill et al, describe a new toxin platform for cancer therapy (Gill et al, 2024).

Diphtheria toxin (DT) produced by *Corynebacterium diphtheriae*, became a first-choice candidate for the development of immunotoxins. DT, a 60 kDa polypeptide, exhibits an ‘AB’ toxin architecture, made of two subunits harboring three distinct domains with a characteristic Y-like shape: the B subunit, responsible for cell receptor binding (R domain) and membrane translocation (T domain), and the A subunit, which orchestrates intracellular catalytic activity (C domain). DT’s mode of action—characterized in depth through decades of investigation—involves binding to host cells, T-mediated translocation of the A subunit into the cytosol, followed by the inhibition of protein synthesis through ADP-ribosylation and subsequent inactivation of eukaryotic elongation factor 2 (eEF-2). Notably, its remarkable toxicity enables a single molecule of DT to lethally impact a cell, rendering it one of the most potent toxins ever discovered (Murphy, 2011). These unique attributes position DT as an ideal candidate for developing cancer-targeting immunotoxins. By replacing the receptor-binding domain with antibodies or other cancer-specific protein ligands, DT can selectively target and kill tumorigenic cells while sparing healthy tissues.

**Figure 1:**
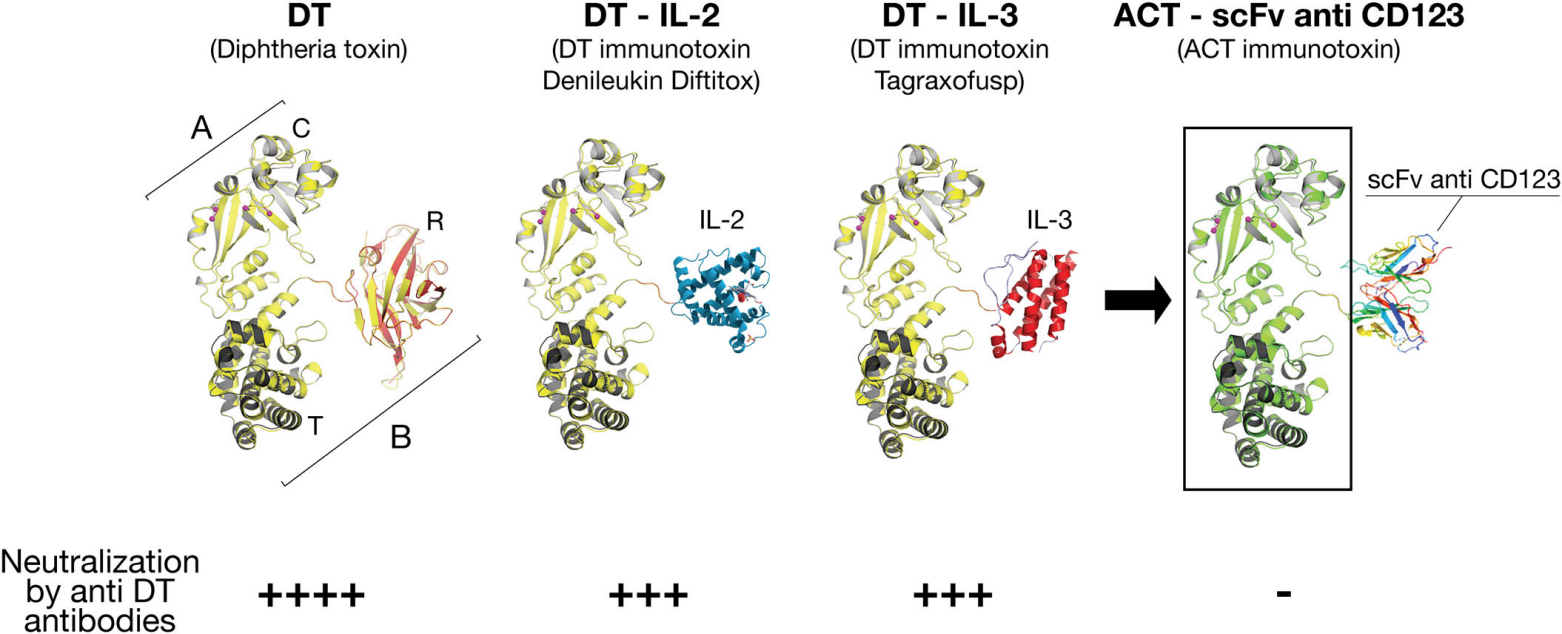
Toxins and immunotoxins. From left to right: structural architecture of DT native toxin; DT-derived immunotoxin denileukin diftitox: the DT receptor-binding domain is replaced by IL-2; DT-derived immunotoxin tagraxofusp: the DT receptor-binding domain is replaced by IL-3; ACT-derived immunotoxin: C and T domains are from ACT, while the R domain is replaced here by scFv against CD123. Affibodies, peptides, or even the homologous ACT domain (SORT-1) could be used for the development of next-generation ACT immunotoxins. Expected effect by anti-DT pre-existing antibodies is also indicated below. Of note: for the purpose of this schematic representation, the 3D structure of DT was used to depict the ACT-based immunotoxins (in green). (Adapted from Malito et al, 2012).

As of today, only two DT-based immunotoxins, Denileukin Diftitox and Tagraxofusp, in which the receptor-binding domains were substituted with IL-2 and IL-3 respectively (Fig. 1), are FDA-approved for clinical use. Indeed, the use of DT-based immunotoxins entails serious limitations. Firstly, “off target toxicity”, which results from DT affecting healthy cells expressing the same target as cancer cells, and neurological toxicity, which results from nonspecific binding to neural tissues, which limit the dosage that can be safely administered. Another and perhaps major limitation of DT-based immunotoxins is linked to the presence of pre-existing anti-DT antibodies in most of the population, induced by the universally recommended vaccination against diphtheria. These antibodies, mostly directed to the translocation domain, reduce the efficiency of DT-based immunotoxins, thus representing a barrier to the broad application of DT-based anti-cancer treatments. Indeed, clinical studies have shown that an inverse correlation exists between pre-existing anti-DT titers and actual drug exposure, underscoring the challenges associated with development of effective DT-based immunotoxins.

An elegant solution that bypasses the presence of anti-DT antibodies consists in mining the microbial pangenome (Medini et al, 2005). With the aim of identifying ancestor species from which Corynebacterium diphtheriae may have evolved, Melnyk and colleagues had previously conducted an extensive bioinformatic search for distant relatives of diphtheria toxin in the GenBank Database containing 177,666 genome sequences (Mansfield et al, 2018). Interestingly, they found genes coding for DT homologs with unique R-binding domains, and distant relatives of the catalytic and translocation domains, suggesting that they encoded for toxins targeting cell receptors in different animal species. One of them was identified in the genome of *Austwickia chelonae*, an ancient reptile pathogen which belongs to the phylum of Actinobacteria and was therefore named Austwickia chelonae toxin (ACT).

In their current study, the authors found that, in spite of the limited sequence conservation, the key amino acids making up the active site (Domenighini and Rappuoli, 1996) were retained in ACT and the three-dimensional structure was similar to DT (Gill et al, 2024). However, given the different R domain, ACT displayed negligible toxicity on mammalian cells. Through an elegant modular chimera experimental approach, the authors found that the functionality of the translocase and catalytic domains of ACT was superior to that of native DT. They used the C and T domains of DT and ACT in parallel to generate new immunotoxins in which the R domains were replaced by single chain variable fragment (scFv) against CD123, by an HER3 targeting affibody, or by a peptide targeting the integrin ανβ63. In all cases, they found that the new ACT-derived immunotoxins displayed a toxicity that was comparable or greater to that of DT-based toxins, while displaying a dramatic decrease of about 6 logs in the susceptibility to pre-existing anti-DT antibodies, in line with the expected low exposure of humans to *Austwickia chelonae* or its toxins.

The discovery of bacterial toxins resistant to neutralization by anti-DT antibodies represents a significant advancement in immunotoxin research. These toxins provide new tools for precision cancer medicine, bringing us closer to the elusive “magic bullet.” In addition, Gill and co-authors highlight the value of exploring the global microbial pangenome, which contains genes from a trillion of microbial species on our planet. In many cases, microbes might already have the solutions that we are looking for.

Apart from serving as scaffolds for immunotoxins, DT derivatives play a crucial role as carrier molecules in bacterial vaccines. For instance, they are widely used in meningococcal and pneumococcal glycoconjugated vaccines (Micoli et al, 2018). However, pre-existing immunity against vaccine carrier proteins can hinder the immune response to antigens conjugated to the same carrier—a phenomenon known as carrier-induced epitopic suppression (CIES) (Pollabauer et al, 2009). Consequently, alternative carrier molecules with DT-like features but unaffected by pre-existing antibodies would be highly desirable. In this context, ACT emerges as an ideal candidate worthy of further exploration.

While the study by Gill et al demonstrates ACT’s utility as a scaffold for new immunotoxins, other applications may thus be envisioned for this molecule in the field of vaccinology.
